# A New Way to Measure the World's Protected Area Coverage

**DOI:** 10.1371/journal.pone.0024707

**Published:** 2011-09-21

**Authors:** Lissa M. Barr, Robert L. Pressey, Richard A. Fuller, Daniel B. Segan, Eve McDonald-Madden, Hugh P. Possingham

**Affiliations:** 1 School of Biological Sciences, University of Queensland, Brisbane, Australia; 2 Australian Research Council Centre of Excellence for Coral Reef Studies, James Cook University, Townsville, Australia; 3 CSIRO Climate Adaptation Flagship and CSIRO Ecosystem Sciences, Dutton Park, Australia; University of Maribor, Slovenia

## Abstract

Protected areas are effective at stopping biodiversity loss, but their placement is constrained by the needs of people. Consequently protected areas are often biased toward areas that are unattractive for other human uses. Current reporting metrics that emphasise the total area protected do not account for this bias. To address this problem we propose that the distribution of protected areas be evaluated with an economic metric used to quantify inequality in income— the Gini coefficient. Using a modified version of this measure we discover that 73% of countries have inequitably protected their biodiversity and that common measures of protected area coverage do not adequately reveal this bias. Used in combination with total percentage protection, the Gini coefficient will improve the effectiveness of reporting on the growth of protected area coverage, paving the way for better representation of the world's biodiversity.

## Introduction

Protected areas are one of the most effective management strategies for abating the rapid decline of biodiversity [1], [2]. Yet despite the recent expansion of the global protected area estate, species extinction rates are not declining; in fact, they are higher than ever [3]. In many cases this is because the designation of protected areas has not been systematic [4], creating an uneven distribution of protection and leaving many vulnerable species [5] and habitats [6], [7] with little or no formal protection.

To reduce this bias in protected area coverage, systematic conservation planning requires that protected area networks be representative of all biodiversity features (e.g. habitats or species) within a region. [4]. Although it is often desirable for threatened components of biodiversity to receive more protection, and thus have higher representation, data are often lacking to define areas that require more protection. In these circumstances, uniform targets, e.g. 10% of every habitat type, are typically used. While achievement of an arbitrary target does not guarantee an adequate reserve system [8], especially at broad scales, it can reduce bias and provide a platform for later expansion of protected areas.

Uniform targets have become a major component of national and international strategies that involve protected areas. For example in 2004, a global policy on biodiversity conservation, the Convention on Biodiversity (CBD), set a target that 10% of each of the world's ecoregions– large areas of land containing geographically distinct assemblages of natural communities [9] –be represented in protected areas. Although uniform targets are commonly used in international policy and the design of protected area networks, there is no current single measure that evaluates equality of protection. The most common measure used to report on protected areas is the percentage of a particular area (e.g. a country) that is protected [10]. Yet reporting only the geographic area under protection obscures how evenly protection is spread across the full range of biodiversity within these areas. Most notably, large areal coverage of a region can be achieved by conserving those habitat types that are cheaper or easier to protect, thus concealing the lack of protection of remaining biodiversity [11], [12].

To address this reporting limitation, the total percentage of area with protection (“total protected”) can be complemented by additional measures such as mean percentage protection of biodiversity features (“mean protected”) [13], [14], or when the uniform target is 10%- the percentage of biodiversity features that have at least 10% protection (“10% or more protected”) [14], [15]. However, these metrics do not provide important information about the distribution of protected areas and can be prone to bias. Mean protected, like total protected, can also be influenced by the protection of large areas not viable for other human uses. While a threshold such as 10% or more protected illustrates how many biodiversity features are protected at or above 10% it gives no further information above or below this target. For example, if a country contained two ecoregions of equal size and protected 9% of the area of each, then 10% or more protected would be zero, despite considerable, and equitable, progress in establishing protected areas. If 90% of one ecoregion but none of the second was protected, then 10% or more protected would be 50%, indicating reasonable progress, despite very uneven protection. To alleviate these reporting problems, a simple additional measure is needed to more effectively illustrate representativeness, particularly as it relates to equality of protection.

Some of the most influential performance measures have arisen from the field of economics. Economic performance measures such Gross Domestic Product (GDP) and Consumer Price Index (CPI) are effective because they are simple, informative for their purpose, and are complemented by other measures that offer a more complete picture of economic performance. Whilst common measures for reporting on protected area coverage are also simple, they are not informative for their purpose because they fail to reveal widespread biases in protection. To address this shortcoming, we propose that the economic metric of inequality, the Gini coefficient [16], be adapted to report protected area coverage.

The Gini coefficient is the most widely known and used measure of inequality in economics [17], [18]. It is derived from the Lorenz curve [19], which is a cumulative distribution function describing inequality. The Gini coefficient measures the difference between a perfectly equitable distribution and the actual distribution of a resource. It is bound between zero (most even) and one (least even), making it easy to interpret. Although originally devised to measure inequality in income distribution, the Gini coefficient has been adapted to disciplines such as health [20], plant biology [21] and, more recently, microcosm studies [22]. It could also be applied to measure how evenly countries or other jurisdictions are protecting their biodiversity, thereby contributing currently missing information on progress towards representative coverage of protected areas.

Here we adapt the Gini coefficient to measure the equality of protection. To do this we have reversed it (1-Gini, “protection equality”) and then converted it to a percentage to be on the same scale as total percentage protection. With our adapted coefficient, one hundred percent is a perfectly equitable distribution of protection and zero percent is completely inequitable. We use protection equality to measure the evenness of protection (by protected areas in IUCN categories I–IV) across the world's terrestrial ecoregions defined by World Wildlife Fund [9] that lie within the boundaries of 83 countries (see Methods for selection process of countries). We demonstrate the utility of protection equality by comparing it to the other three commonly used measures used to assess protected area coverage: total protected, mean protected and 10% or more protected.

## Results

Of the 83 countries analysed, 61 had protection equality <50% (Table S1). Only 3 countries had protection equality >75% and, of these, only Botswana had more than 10% of its total area protected.

**Figure 1:**
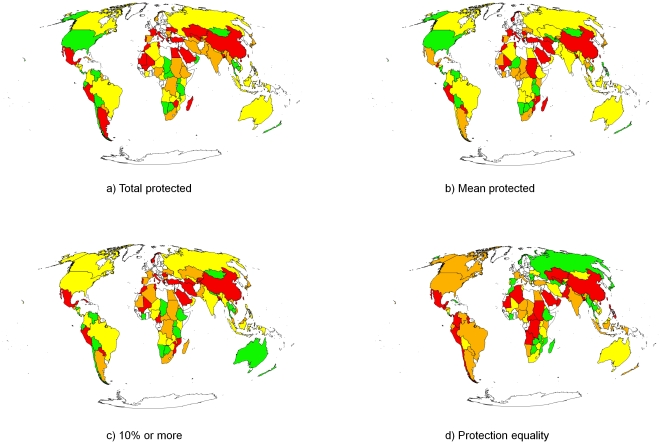
Four measures of protected area coverage in 83 countries analysed. a) total percentage of land protected, b) mean percentage protection of ecoregions, c) percentage of ecoregions with at least 10% protection, d) protection equality. We divided all countries into quartiles for each measure and assigned colours to each quartile: green = highest quartile, yellow = second highest quartile, orange = second lowest quartile, red = lowest quartile.

There was enormous geographic variation in the performance of countries according to the commonly used measures and protection equality (Figure 1). In the Americas many countries were ranked in the first or second quartile for total protected, mean protected and 10% or more protected. However, in terms of protection equality, most of these countries appeared in the third or fourth quartiles (Figure 1). Many African countries performed poorly in all measures, although a few, such as Botswana, Tanzania, Uganda and Zambia were in the top two quartiles for all metrics (Figure 1). In Europe, all countries were ranked in the bottom two quartiles for total protected, mean protected and 10% or more protected. However, for protection equality more than 77% of European countries ranked in the top two quartiles (Figure 1). In Asia, Russia was in the top two quartiles for all measures, while China was in the bottom quartile for all.

The three commonly used reporting metrics provided similar information across the 83 countries. Many countries in the top two quartiles for mean protected were also in the top two quartiles for total protected (74%) and 10% or more protected (83%). Most countries in the top half for 10% or more protected were also in the top half for total protected (85%). Mean protected and 10% or more protected are both positively correlated with total protected (τ = 0.598, p<0.001, τ = 0.608, p<0.001, respectively) and with each other (τ = 0.685, p<0.001) (n = 83 for all).

In contrast, protection equality was poorly correlated with values for the other metrics. Of the countries ranked in the top two quartiles for protection equality, only 41% were also in the top two quartiles for mean protected, while 46% were in the top two quartiles for 10% or more protected and total protected. There was no significant correlation between protection equality and the other three metrics (τ = 0.0790, p>0.28 for total protected; τ = 0.019, p>0.8 for mean protected; and τ = 0.064, p>0.4 for 10% or more protected, all n = 83).

## Discussion

Our results indicate that a protection equality measure could help to overcome many of the current limitations of reporting protected area coverage. Most countries have an unevenly distributed coverage of protected areas, which is not illustrated by the three commonly used metrics. Moreover, all metrics other than protection equality were strongly correlated with each other, indicating substantial redundancy.

Generally, countries that performed well according to the three common metrics of protected area system performance did not perform well in protection equality. This could arise from reservation bias toward areas that are not useful for extractive uses. For example, the United States has protected more than 10% of its land mass, yet was in the bottom quartile for protection equality because protection in that country is heavily biased to higher elevations and less productive soils [23]. Protection in many other countries is also biased to high altitude or steep areas that are difficult to access [24].

Low protection equality could also result from the protection of ecoregions that are the most threatened or globally iconic. This is probably the case in Brazil, in which most of the globally important Amazon rainforest occurs [25]. Since 2003, most of the area added to the protected area system globally has been in the Brazilian section of the Amazon [15], perhaps explaining why Brazil was ranked highly by the three commonly used metrics but not by protection equality. However in Brazil not all areas needing protection are receiving it. The Cerrado biodiversity hotspot is more threatened than the Amazon [26], yet only 2% of its area is protected, despite having a higher deforestation rate than the Amazon [27]. Therefore, while Brazil may be doing well in protecting a globally iconic ecoregion, other ecoregions are not receiving similar attention, which is revealed by the protection equality metric.

Ecoregions are a useful surrogate for biodiversity in global analyses because they are consistent across the world. Although an ecoregional analysis allowed us to make a globally coherent comparison among countries, ecoregions are large, heterogeneous units and might produce different values of protection equality within and between countries than finer ecosystem classifications. Consequently, when evaluating protection equality at a national level, the analysis should focus on the biodiversity surrogates typically used for protected area planning within the countries concerned.

We propose that measures of protection equality are not used in isolation, but rather in combination with one of the existing protection metrics. Importantly, protection equality does not reflect how much overall protection has been achieved, which means that a country could achieve high protection equality by protecting just 1% of its land mass, distributed evenly across ecoregions. In our analyses, many countries scored well in protection equality because of low overall protection. Therefore, as a baseline for reporting on protected area coverage, we propose that protection equality be paired with total percentage protected because, together, they reflect both the amount of overall protection and the evenness of this protection. Ideally, countries that use uniform targets should be aiming towards increasing protected area coverage, while also maintaining or increasing protection equality.

The importance of representativeness has long been recognized as a key principle for conservation planning [4]. Incorporating this principle into international treaties, such as the CBD, also establishes its importance for global policy. Despite this, the metrics most frequently used to report on protected area networks ignore a key aspect of representativeness and, in some instances, overestimate progress towards it. This failure to align the objectives of conservation with appropriate reporting measures can mislead decision makers and the public alike, and eventually undermine further expansion of protected areas. If countries are to evaluate real progress towards achieving a representative network of protected areas, then reporting metrics that more accurately align with conservation principles, such as protection equality, are urgently needed.

## Methods

We used the refined map of 825 ecoregions developed by the World Wildlife Fund [9], [28] to represent the variety of biodiversity in countries because they have been consistently mapped globally. We excluded ‘Lakes’ and ‘Rock and Ice’, leaving 823 ecoregions that could be mapped across countries. To account for spatial mismatches between country boundaries and ecoregions, we removed all mangrove ecoregions and also ecoregions overlapping countries that were smaller than 100 km^2^ and less than 1% of the total ecoregion area. To permit meaningful estimates of protection equality, countries were retained for analysis if: (i) they contained at least five ecoregions; and (ii) they provided at least 1% protection for at least one ecoregion. This left a total of 83 countries within which we could compare protection equality to the other three commonly used metrics for protected area coverage: total percentage of area protected (total protected), mean percentage of ecoregion area protected (mean protected), and percentage of ecoregions with at least 10% protected (10% or more protected).

To estimate protected area coverage, we used 2009 data from the World Database on Protected Areas [29]. Protected areas fall under two broad IUCN management themes: management primarily for biodiversity (categories I–IV), or biodiversity conservation combined with sustainable use (categories V and VI). We excluded categories V and VI and used only protected areas managed primarily for biodiversity (I–IV). We included all national and international protected areas, but excluded those that were not officially ‘designated’ (e.g. voluntary protected areas and those that are recommended rather than established). We created circular buffers for protected areas for which only point locations and estimated extents were available. We set the radii of these protected areas to produce circles with areas equal to reported total areas. Although this allowed us to include more protected areas, circular features do not reflect actual boundaries, and this would have caused some over- and under-estimation of percentages of ecoregions protected. This is considered a minor effect at the ecoregional scale but could have more influence at finer resolutions [15]. Protection in Europe is likely to be underestimated because NATURA 2000 protected area data were not available.

Gini coefficients for each country were derived from Lorenz curves, which are cumulative distribution functions used to describe inequality [19]. To calculate Lorenz curves for each country, the percentage protection of ecoregions were ranked smallest to largest and the cumulative proportion of protection was calculated and then plotted against the cumulative percentage of ecoregions. If protection is distributed equally amongst all ecoregions (e.g. each ecoregion has exactly 10% protection) then the Lorenz curve will form the line of equality (Figure S1). If there is inequality in protection across ecoregions then the Lorenz curve will lie below the line of equality (Figure S1). The Gini coefficient expresses the area between the line of equality and the Lorenz curve.

We calculated Gini coefficients using Brown's formula [20]:
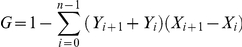

*X_i_*: cumulative proportion of the area of n ecoregions, for *i* = 1,…,n

*Y_i_*: cumulative proportion of the area of protection of n ecoregions, for *i* = 1,…,n

We calculated correlations between measures of protected area coverage with Kendall's correlation coefficient (τ) because the data were not normally distributed.

## Supporting Information

Figure S1 **The Lorenz curve and Gini coefficient.** Cumulative percentage of protection is the percentage of protection that belongs to each ecoregion, while cumulative percentage of ecoregion illustrates the proportion of the total area that ecoregion represents.
(DOC)

Table S1 **Protection equality (%) values for 83 countries analysed.** n = number of ecoregions analysed in each country.
(DOC)

## References

[1] Mulongoy KJ, Chape S (2004).

[2] Possingham HP, Wilson KA, Andelman SJ, Vynne CH, Groom MJ, Meffe GK, Carroll CR (2006). Protected Areas: Goals, limitations and designs.. Principles of Conservation Biology. 3rd ed.

[3] World Wildlife Fund (2006). Living Planet Report 2006; Nature WWF, editor.

[4] Margules CR, Pressey RL (2000). Systematic conservation planning.. Nature.

[5] Rodrigues ASL, Andelman SJ, Bakarr MI, Boitani L, Brooks TM (2004). Effectiveness of the global protected area network in representing species diversity.. Nature.

[6] Hoekstra JM, Boucher TM, Ricketts TH, Roberts C (2005). Confronting a biome crisis: global disparities of habitat loss and protection.. Ecology Letters.

[7] Fuller RA, McDonald-Madden E, Wilson KA, Carwardine J, Grantham HS (2010). Replacing underperforming protected areas achieves better conservation outcomes.. Nature.

[8] Pressey RL, Cowling RM, Rouget M (2003). Formulating conservation targets for biodiversity pattern and process in the Cape Floristic Region, South Africa.. Biological Conservation.

[9] Olson DM, Dinerstein E, Wikramanayake ED, Burgess ND, Powell GVN (2001). Terrestrial Ecoregions of the World: A New Map of Life on Earth.. BioScience.

[10] 2010 Biodiversity Indicators Partnership (2009).

[11] Pressey RL (1994). Ad Hoc Reservations: Forward or Backward Steps in Developing Representative Reserve Systems?. Conservation Biology.

[12] Pressey RL, Hager TC, Ryan KM, Schwarz J, Wall S (2000). Using abiotic data for conservation assessments over extensive regions: quantitative methods applied across New South Wales, Australia.. Biological Conservation.

[13] Coad L, Burgess NB, Fish L, Ravillious C, Corrigan C (2008). Progress towards the Convention on Biological Diversity terrestrial 2010 and marine 2012 targets for protected area coverage.. Parks.

[14] UNEP-WCMC (2008). State of the world's protected areas: an annual review of global conservation progress.

[15] Jenkins C, Joppa L (2009). Expansion of the global terrestrial protected area system.. Biological Conservation.

[16] Gini C (1921). Measurement of Inequality and Incomes.. The Economic Journal.

[17] Allison P (1978). Measures of inequality.. American Sociological Review.

[18] Coulter PB (1989). Measuring Inequality: A methodological handbook.

[19] Lorenz MO (1905). Methods of Measuring the Concentration of Wealth.. Publications of the American Statistical Association.

[20] Brown M (1994). Using Gini-Style Indices to Evaluate the Spatial Patterns of Health Practitioners: Theoretical Considerations and an Application Based on Alberta Data.. Social Science Medicine.

[21] Damgaard C, Weiner J (2000). Describing Inequality in Plant Size or Fecundity.. Ecology.

[22] Wittebolle L, Marzorati M, Clement L, Balloi A, Daffonchio D (2009). Initial community evenness favours functionality under selective stress.. Nature.

[23] Scott JM, Davis FW, McGhie G, Wright RG, Groves C (2001). Nature Reserves: Do They Capture the Full Range of America's Biological Diversity?. Ecological Applications.

[24] Joppa L, Pfaff A (2009). High and far: biases in the location of protected areas.. PLoS ONE.

[25] Olson DM, Dinerstein E (1998). The global 200: a representation approach to conserving the Earth's most biologically valuable ecoregions.. Conservation Biology.

[26] Myers N, Mittermeier RA, Mittermeier CG, da Fonseca GAB, Kent J (2000). Biodiversity hotspots for conservation priorities.. Nature.

[27] Klink CA, Machado RB (2005). Conservation of the Brazilian Cerrado.. Conservation Biology.

[28] World Wildlife Fund (2008). Terrestrial ecoregions of the world.

[29] World Database of Protected Areas (2009). World Database on Protected Areas (WDPA) Annual Release 2009.

