# Supplementary figures and images for: A New Way to Measure the World's Protected Area Coverage
Source: PLoS One. 2011 Sep 21;6(9):e24707. doi: 10.1371/journal.pone.0024707 (PMC3177831; doi:10.1371/journal.pone.0024707)

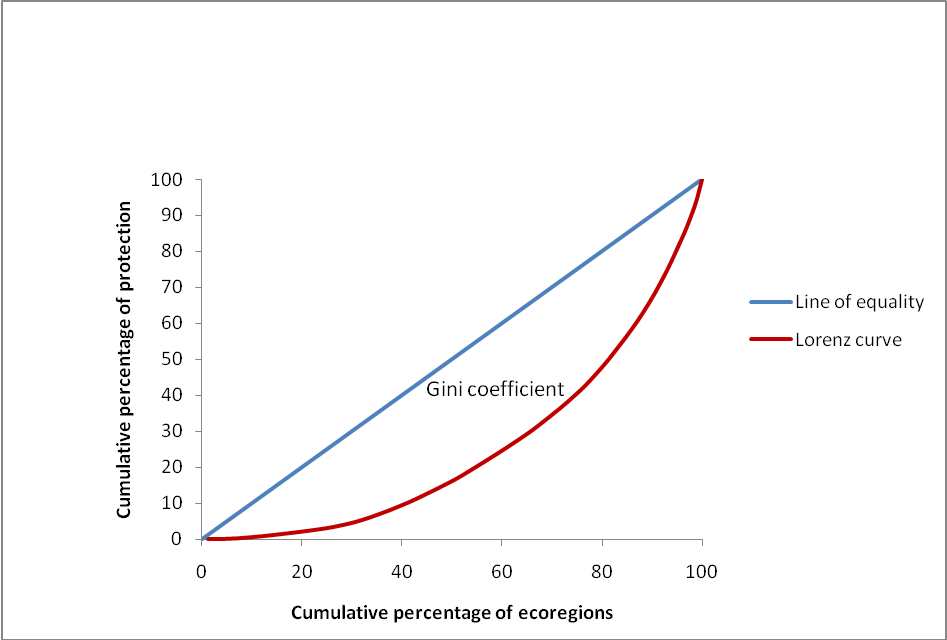

Supplement: Figure S1 — The Lorenz curve and Gini coefficient. Cumulative percentage of protection is the percentage of protection that belongs to each ecoregion, while cumulative percentage of ecoregion illustrates the proportion of the total area that ecoregion represents. (DOC) [file pone.0024707.s001.doc]
