# Supplementary material for: A New Way to Measure the World's Protected Area Coverage
Source: PLoS One. 2011 Sep 21;6(9):e24707. doi: 10.1371/journal.pone.0024707 (PMC3177831; doi:10.1371/journal.pone.0024707)
Supplement: Table S1 — Protection equality (%) values for 83 countries analysed. n = number of ecoregions analysed in each country. (DOC) [file pone.0024707.s002.doc]

| **Country** | **Protection Equality** | **n** |
| --- | --- | --- |
| Algeria | 16.87 | 8 |
| Angola | 28.27 | 15 |
| Argentina | 32.51 | 17 |
| Australia | 50.86 | 38 |
| Bangladesh | 18.08 | 6 |
| Bhutan | 73.61 | 11 |
| Bolivia | 42.71 | 16 |
| Botswana | 77.98 | 6 |
| Brazil | 34.38 | 46 |
| Bulgaria | 61.53 | 6 |
| Cambodia | 44.65 | 7 |
| Cameroon | 47.90 | 11 |
| Canada | 34.58 | 51 |
| Central African Republic | 26.73 | 6 |
| Chad | 40.94 | 7 |
| Chile | 29.72 | 9 |
| China | 1.96 | 61 |
| Colombia | 21.60 | 30 |
| Congo, DRC | 19.50 | 17 |
| Costa Rica | 55.10 | 5 |
| Cote d'Ivoire | 58.62 | 5 |
| Cuba | 74.73 | 5 |
| Ecuador | 39.39 | 10 |
| Egypt | 32.39 | 11 |
| Eritrea | 47.38 | 7 |
| Ethiopia | 57.56 | 11 |
| France | 39.24 | 9 |
| Greece | 44.35 | 6 |
| Guatemala | 13.89 | 10 |
| Guyana | 19.65 | 10 |
| Honduras | 36.68 | 5 |
| India | 33.94 | 48 |
| Indonesia | 36.05 | 36 |
| Iran | 52.52 | 18 |
| Italy | 32.81 | 9 |
| Japan | 54.23 | 8 |
| Kazakhstan | 29.37 | 19 |
| Kenya | 39.38 | 11 |
| Kyrgyzstan | 47.57 | 8 |
| Madagascar | 56.00 | 6 |
| Malawi | 46.22 | 8 |
| Malaysia | 42.64 | 9 |

| **Country** | **Protection Equality** | **n** |
| --- | --- | --- |
| Mali | 43.31 | 6 |
| Mauritania | 14.39 | 8 |
| Mexico | 23.33 | 45 |
| Mongolia | 45.15 | 16 |
| Morocco | 80.87 | 6 |
| Mozambique | 64.02 | 11 |
| Myanmar | 76.11 | 17 |
| Namibia | 33.60 | 12 |
| Nepal | 41.29 | 11 |
| New Zealand | 41.89 | 12 |
| Nicaragua | 46.20 | 7 |
| Niger | 38.86 | 6 |
| Nigeria | 34.75 | 13 |
| Norway | 56.69 | 5 |
| Oman | 27.73 | 6 |
| Pakistan | 24.49 | 17 |
| Panama | 32.26 | 9 |
| Paraguay | 39.26 | 7 |
| Peru | 25.74 | 17 |
| Philippines | 29.26 | 9 |
| Portugal | 45.29 | 6 |
| Romania | 72.82 | 6 |
| Russia | 61.19 | 49 |
| Saudi Arabia | 25.39 | 6 |
| South Africa | 46.75 | 16 |
| Spain | 61.30 | 10 |
| Sudan | 15.87 | 17 |
| Tajikistan | 69.54 | 6 |
| Tanzania | 46.29 | 16 |
| Thailand | 58.12 | 13 |
| Tunisia | 45.50 | 5 |
| Turkey | 37.13 | 14 |
| Turkmenistan | 55.05 | 8 |
| Uganda | 49.20 | 8 |
| Ukraine | 43.42 | 6 |
| United States | 32.71 | 91 |
| Uzbekistan | 25.29 | 7 |
| Venezuela | 41.57 | 24 |
| Vietnam | 46.71 | 12 |
| Zambia | 70.12 | 9 |
| Zimbabwe | 44.81 | 6 |
